# Electrochemical Detection of Prostate Cancer—Associated miRNA-141 Using a Low-Cost Disposable Biosensor

**DOI:** 10.3390/bios15060364

**Published:** 2025-06-06

**Authors:** Alexander Hunt, Gymama Slaughter

**Affiliations:** 1 Center for Bioelectronics, Old Dominion University, Norfolk, VA 23508, USA; 2 Department of Electrical and Computer Engineering, Old Dominion University, Norfolk, VA 23508, USA

**Keywords:** prostate cancer, paper-based electrochemical biosensor, gold inkjet printing, microRNA-141 detection, synthetic urine

## Abstract

Prostate cancer is the second leading cause of cancer-related deaths among men in the United States. The early detection of aggressive forms is critical. Current diagnostic methods, including PSA testing and biopsies, are invasive and often yield false results. MicroRNA-141 (miRNA-141) has emerged as a promising non-invasive biomarker due to its elevated levels in the urine of patients with metastatic prostate cancer. Here, a low-cost, paper-based electrochemical biosensor for the sensitive detection of miRNA-141 in synthetic urine is reported. The device employs inkjet-printed gold electrodes on photopaper, functionalized with thiolated single-stranded DNA-141 capture probes for specific target recognition. The biosensor achieves a sensitivity of 78.66 fM µA^−1^ cm^−2^ and a linear detection range of 1 fM to 100 nM, encompassing clinically relevant concentrations of miRNA-141 found in patients with metastatic prostate cancer. A low limit of detection of 2.15 fM, strong selectivity against non-target sequences, and a rapid response time of 15 min further highlight the diagnostic potential of the device. This platform represents a significant advancement in the development of point-of-care diagnostic tools for prostate cancer and is readily adaptable for detecting other disease-specific miRNAs through simple probe modification. As such, it holds broad promise for accessible, early-stage cancer detection and longitudinal disease monitoring in diverse clinical settings.

## 1. Introduction

Prostate cancer remains the second most diagnosed cancer and second leading cause of cancer deaths of men in the United States [1]. According to the CDC, there were 236,659 reported new cases of prostate cancer and 32,563 reported deaths in 2021. The 5-year survival rate for prostate cancer is significantly higher (almost 100%) when the cancer is localized (confined to the prostate gland) compared to when it has metastasized (dropping to around 33%) [2]. Despite significant advancements in prostate cancer detection and monitoring, current screening and diagnostic methods for prostate cancer, such as prostate specific antigen (PSA) blood testing, digital rectal examination (DRE), and biopsies, present several limitations [3]. PSA testing lacks specificity, often yielding false negatives and false positives that result in unnecessary biopsies for patients, and PSA cannot be quantified to distinguish between indolent and aggressive cancer types [4]. DRE is invasive and unreliable for determining benign from cancerous growths [5]. Although the only standard for prostate cancer confirmation, biopsies are highly invasive, carry risks of infection, bleeding, and erectile dysfunction, and suffer from sampling errors due to the heterogeneous nature of tumors [6]. Given the highly invasive screening and diagnostic procedures, the unreliability of PSA blood levels, and the extent of side effects that are attributed to biopsies, men are highly discouraged from and often avoid initial and follow-up screenings. Therefore, the identification of new biomarkers that can be obtained non-invasively for early prostate cancer detection and disease progression monitoring is needed to prevent unwarranted death.

MicroRNAs (miRNAs) are small (19–25 nucleotides), non-coding RNAs that regulate gene expression by mRNA degradation or translation inhibition [7]. It has been shown that miRNAs are produced at abnormal levels within malignant cells and can be quantified to differentiate indolent and aggressive prostate cancer [8]. Their dysregulation classifies them as either oncogenic miRNAs (onco-miRs), when overproduced, or tumor-suppressive miRNAs (TS-miRs), when underproduced [9,10]. Moreover, miRNAs show great potential as prostate cancer diagnostic biomarkers due to their presence in easily accessible body fluids, including blood and urine [11].

Urine serves as an advantageous reservoir for cancer-related biomarkers, as it contains tumor-derived proteins, nucleic acids (including prostate cancer-related miRNAs), extracellular vesicles, and cellular debris [12]. This rich biomolecular environment makes urine a valuable medium for non-invasive prostate cancer detection, given its direct connection to the prostate [13].

A major clinical concern in prostate cancer is bone metastasis, which remains a leading cause of mortality in patients with advanced disease [14]. The absence of reliable biomarkers for the early detection of bone metastases presents a significant challenge in treatment. However, recent studies suggest that miRNA analysis from urine may aid in the early identification of metastatic progression, offering a promising avenue for improving clinical outcomes [15]. Notably, miRNA-141 has been shown to enhance prostate cancer progression, particularly in bone metastasis, by regulating osteoblast activity and influencing bone remodeling [16]. Elevated miRNA-141 levels in urine are linked to increased metastatic bone lesions, high Gleason scores, lymph node metastases, and castration resistance [17]. Its upregulation in metastatic prostate cancer patients and cell lines after castration suggests a role in androgen regulation, and it can promote cell growth by regulating Kruppel-like factor 9 [18,19]. However, it has also been shown that miRNA-141 acts as a TS-miR by targeting key metastasis-related genes, including CD44, EZH2, and Rho GTPases, inhibiting cell growth, migration, and invasion [20]. It also suppresses the NF-κB pathway, reducing invasion and bone metastasis [21]. Therefore, quantifying significantly high or low levels of miRNA-141 may provide insights into whether prostate cancer metastasizes to the bone.

The inherent characteristics of miRNAs, in general, such as short length, sequence homology, and possible low abundance, pose formidable challenges for the development of detection methodologies [22]. Traditional detection methods, including Northern blotting, quantitative reverse transcription polymerase chain reaction (RT-qPCR), and microarrays, exhibit low sensitivity, protracted procedures, inadequate specificity, and the necessity of sophisticated equipment [22], which limits their practical application in clinical settings [23]. To address these issues, electrochemical biosensors have gained momentum as viable alternatives for miRNA detection due to their convenient usability, high sensitivity, rapid response times, and potential for miniaturization [24]. Importantly, the choice of electrode substrate material and fabrication technique significantly impacts sensor performance, cost, and applicability in real-world settings [25].

Compared to classical electrodes, paper-based substrates offer several advantages, as they are cost-effective, readily accessible, and disposable, making them ideal for point-of-care (POC) applications, especially in resource-limited settings [26,27,28]. While traditional ceramic substrates provide excellent stability, they suffer from limited design flexibility and uneven surface morphologies [29]. Similarly, glassy carbon electrodes offer good conductivity but require extensive surface treatment and lack flexibility [30]. Laser-induced graphene (LIG) substrates, though cost-effective and scalable, often exhibit structural variability and degradation over time, leading to inconsistent sensor performance [31]. In contrast, paper-based electrodes enable simplified fabrication and integration with microfluidics, making them attractive for portable diagnostics [32]. The most common fabrication techniques for paper-based electrodes are screen printing and inkjet printing [33]. Screen-printing electrodes is a process that presses ink through a stencil mask into a particular pattern [34]. The major limitations of screen printing on paper electrodes include the difficulty of rapid mass-production on flexible substrates and the low resolution of screen printing compared to other printing techniques [35]. On the other hand, inkjet printing offers both high resolution and the ability to rapidly mass-produce electrodes by directly printing a design onto the paper substrate [36].

Herein, we developed a fully functioning gold inkjet-printed electrochemical system on photopaper that quantifies miRNA-141 levels in synthetic urine relevant for advanced prostate cancer detection and monitoring. The development of a paper-based gold electrode array that leverages an inkjet printing technique on photopaper showcases the applicability of a rapid and large-scale fabrication procedure. The biosensor uses thiolated self-assembled monolayers to immobilize single-stranded DNA (ssDNA) onto gold nanoparticles (AuNPs) electrodeposited on the printed gold surface. As previously reported [37], we utilized AuNP electrodeposition to increase the electroactive surface area, improve ssDNA probe conjugation efficiency, and fill post-sintering insulation gaps. As a result, the specific capture of miRNA-141 in synthetic urine was facilitated, and the electronic signal was amplified to achieve a sensitivity of 78.66 fM µA^−1^ cm^−2^. Moreover, the biosensor exhibited a broad linear detection range from 1 fM to 100 nM and a rapid response time of 15 min. Our biosensor demonstrates a low limit of detection (LOD) of 2.15 fM miRNA-141 in synthetic urine and excellent selectivity against interferent species. To the best of our knowledge, this is the first report of an inkjet-printed gold three-electrode system on photopaper applied to the electrochemical detection of miRNA-141 biomarkers. The proposed biosensor platform can be easily adapted to detect a wide range of cancer-related and disease-associated miRNAs by modifying the capture probes on the electrode surface. This versatility and our fabrication design enable the mass-production of diagnostic tools that are non-invasive, low-cost, and disposable, empowering limited-resource settings and ultimately transforming patient outcomes on a global scale.

## 2. Materials and Methods

### 2.1. Chemicals and Solutions

Sulfuric acid (H_2_SO_4_), sodium chloride (NaCl), sodium hydroxide (NaOH), and fetal bovine serum (FBS) were purchased from Thermo Fisher Scientific, Waltham, MA, USA. Potassium ferricyanide (K_3_[Fe(CN)_6_]), gold (III) chloride trihydrate (HAuCl_4_·3H_2_O), potassium chloride (KCl), potassium phosphate monobasic (KH_2_PO_4_), sodium phosphate dibasic (Na_2_HPO_4_), ethylenediaminetetraacetic acid (EDTA), tris(2-carboxyethyl)phosphine (TCEP), sodium sulfate (Na_2_SO_4_), ammonium chloride (NH_4_Cl), calcium chloride (CaCl_2_), and urea were purchased from Sigma-Aldrich, Saint Louis, MO, USA. Tris hydrochloride (Tris-HCl) was purchased from Amresco, Inc., Solon, OH, USA. All oligonucleotide sequences (Table 1) were purchased from Integrated DNA Technologies (IDT), Coralville, IA, USA. The JG-106 gold ink (nanoparticle size of 30–50 nm) was purchased from Novacentrix, Round Rock, TX, USA. The polyimide (PI) tape was purchased from MYJOR, Shenzhen China. Silver/silver chloride (Ag/AgCl) paste was purchased from CH Instrument, Inc., Austin, TX, USA. All solutions were prepared using deionized water (18.2 MΩ cm^2^). A 0.01 M phosphate-buffered saline solution with a pH of 7.4 (1X PBS) was prepared with 137 mM NaCl, 2.7 mM KCl, 1.8 mM KH_2_PO_4_, and 10 mM Na_2_HPO_4_. A 1X Tris-HCl EDTA (TE) buffer solution with a pH of 8.0 was prepared with 10 mM Tris-HCl and 1 mM EDTA using drops of 10 M NaOH to adjust the pH to 8.0. The synthetic urine solution was prepared according to Shinde et al. (i.e., 1.663 mM CaCl_2_, 30.95 mM KCl, 30.05 mM NaCl, 11.96 mM Na_2_SO_4_, 23.667 mM NH_4_Cl, and 249.750 mM urea [38]), with an addition of FBS diluted 1000-fold. TCEP and ssDNA-141 solutions were prepared in 1X PBS.

### 2.2. Apparatus and Instrumentation

The Dimatix Materials Printer DMP-2850 (Fujifilm, Inc., Tokyo, Japan) was employed to print gold-integrated electrodes on photopaper. The electrodeposition of AuNPs was carried out using direct current potential amperometry (DCPA) with the EC Epsilon EClipse™ Potentiostat (Bioanalytical Systems, Inc., West Lafayette, IN, USA), employing external Ag/AgCl reference and platinum wire counter electrodes. For electrochemical testing, Ag/AgCl paste was used for the reference electrode fabrication, and the gold ink electrode was used as the counter electrode. Cyclic voltammetry (CV) was performed within a potential range from −0.1 to 0.4 V at a scan rate of 100 mV s^−1^, and square wave voltammetry (SWV) with a potential range from −0.1 to 0.3 V, a potential step of 1 mV, an amplitude of 40 mV, and a frequency of 4 Hz was conducted to quantitatively assess the ssDNA-141 to miRNA-141 hybridization events. All electrochemical tests were performed in triplicate.

### 2.3. Inkjet Printing Parameters of the 48-Electrode System Array

The 48-electrode system array was designed using CoralDRAW (2019 (21)). The working, counter, and reference electrodes were defined with a 1.5 mm thickness with gaps of 1 mm. The circular working electrode area was defined with a 3 mm diameter. The .bmp file was exported to the DMP-2850 inkjet printer to be converted to a .ptf file for printing. Double-sided tape was used to secure the photopaper onto the printer platen. The software was then configured with a substrate thickness (photopaper and double-sided tape) of 670 µm and a jetting speed of 50 Hz for printing the electrode array. In total, 1 mL of gold ink was injected into a cartridge tank, followed by capping the cartridge tank with a DMC-11610 (10 pL drop-size) cartridge head. The platen and ink cartridge temperatures were set at 45 °C and 28 °C, respectively. All 16 jets were used during printing with a resolution of 1016 DPI and a jetting voltage of 25 V. Six layers of gold ink were printed onto the photopaper to fabricate the 48-electrode system array (Figure S1A, Supplementary Materials).

### 2.4. Fabrication of Gold Inkjet-Printed Paper Electrodes (GIPEs)

**Figure 1:**
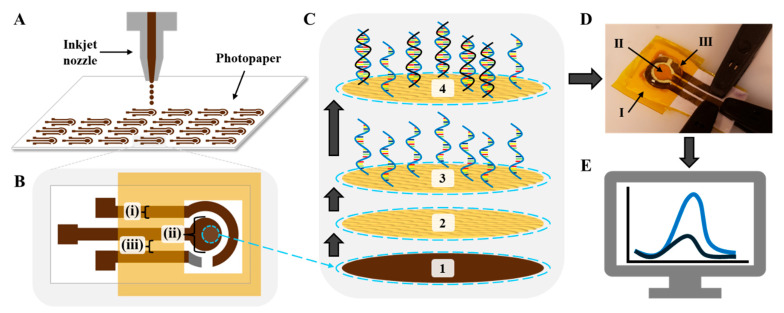
Schematic illustration of the GIPE system fabrication and sensing mechanism. (**A**) Inkjet printing of the 48-electrode system array. (**B**) GIPE: (i) 1.5 mm trace width; (ii) 3 mm diameter working electrode; (iii) 1 mm gaps between traces. (**C**) Fabrication of sensing platform: (1) gold ink working electrode; (2) working electrode after AuNP electrodeposition; (3) after ssDNA-141 capture probe immobilization; (4) after miRNA-141 hybridization. (**D**) Photograph of electro-analytical setup with (K_3_Fe(CN)_6_^4−/3−^) electrolyte solution and interrogation via BASi potentiostat: (I) PI tape lamination; (II) sensing platform; (III) Ag/AgCl paste reference electrode. (**E**) Voltammogram response representation: blue curve is current response of vacant ssDNA-141 capture probes; black curve is decreased current response due to target miRNA-141 hybridization.

Gold inkjet-printed paper electrodes (GIPEs) were fabricated. Following inkjet printing, electrode arrays were air-dried overnight in a chemical fume hood at room temperature and subsequently sintered at 140 °C for 1 h in a conventional laboratory oven to ensure ink adhesion and conductivity. Individual electrodes (Figure 1A) were then cut from the array and laminated with polyimide (PI) tape (Figure 1B). The PI tape lamination forms a containment area to define the working surface area and house the electrolyte during testing. Importantly, the water-resistant gloss of the photopaper prevents the solutions applied to the working surface from wicking throughout the photopaper. Prior to modification, electrochemical cleaning was performed using cyclic voltammetry (CV) in 0.05 M H_2_SO_4_ for 15 cycles over a potential range of -0.1 V to 1.5 V at a scan rate of 50 mV s^−1^. The electrodes were rinsed thoroughly with DI water and dried in a desiccator. AuNPs were electrodeposited onto the working electrode surface using chronoamperometry in a 2 mM HAuCl_4_ solution with Ag/AgCl as the reference and a platinum wire as the counter electrode. Deposition was performed at ࢤ0.5 V for 1 h, yielding the GIPE/AuNPs configuration (Figure 1C(2)). The modified electrodes underwent a second CV-based cleaning step under identical conditions, followed by DI water rinsing and desiccation. The reference electrode on the GIPE was fabricated by applying Ag/AgCl paste and chemically chlorinating it with 10 μL of household bleach for 5 min at room temperature. A schematic of the complete fabrication process and sensing configuration is shown in Figure 1, with additional fabrication details provided in Figure S1B (Supplementary Materials).

### 2.5. Immobilization of ssDNA-141 Probe on GIPE/AuNPs and miRNA-141 Hybridization

To enable the selective detection of miRNA-141, a thiolated ssDNA probe complementary to miRNA-141 (ssDNA-141) was immobilized onto the surface of the GIPE/AuNPs working electrode. Prior to immobilization, the disulfide bonds of the thiolated probe were reduced using tris(2-carboxyethyl)phosphine (TCEP) at a molar ratio of 100:1 (TCEP:ssDNA-141). The solution was vortexed vigorously for one minute every 15 min over a total duration of one hour at room temperature. The activated probe solution was then stored at 4 °C until use. For probe immobilization, 3 μL of the TCEP-treated ssDNA-141 solution was drop-cast directly onto the working electrode area of the GIPE/AuNPs. The modified electrodes were incubated in a sealed humidity chamber at room temperature for 3 h to allow for stable thiol–gold bond formation between the probe and the AuNP surface. After incubation, the electrodes (now designated as GIPE/AuNPs/ssDNA) were rinsed thoroughly with TE buffer to remove unbound ssDNA and residual reagents. To preserve probe integrity and prevent denaturation, the electrodes were stored in the humidity chamber at 4 °C, with TE buffer covering the electroactive surface until further analysis.

To evaluate the detection performance of the fabricated biosensor, hybridization experiments were conducted using miRNA-141 target solutions prepared in either 1X PBS or synthetic urine. Prior to each measurement, the GIPE/AuNPs/ssDNA-modified electrode was gently rinsed with TE buffer to remove loosely bound surface contaminants. Excess buffer was carefully aspirated from the electrode surface without disturbing the working area. A 50 μL aliquot of miRNA-141 target solution at a specified concentration (ranging from 1 fM to 100 nM) was then dispensed onto the working surface of the GIPE/AuNPs/ssDNA electrode. The sample droplet was confined within a hydrophilic containment well defined by the PI tape lamination. The electrode was incubated at room temperature for 15 min to facilitate hybridization between the immobilized ssDNA-141 probe and the complementary miRNA-141 target. After incubation, the hybridized sample was removed by pipetting, and the electrode surface was subjected to a rigorous washing protocol to eliminate unbound or nonspecifically adsorbed miRNA. Specifically, five sequential washing steps were performed using 100 μL of TE buffer. Each wash involved the addition and removal of the buffer by pipette. This five-step washing cycle was then repeated once to ensure the complete removal of residual target molecules.

## 3. Results and Discussion

### 3.1. Electrochemical Characterization of GIPE Surface Modification

**Figure 2:**
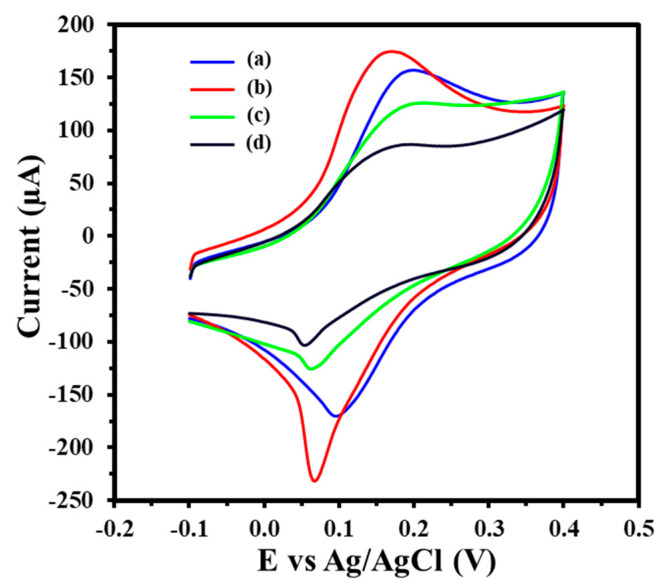
CV of GIPE (a), GIPE/AuNPs (b), GIPE/AuNPs/ssDNA (c), and GIPE/AuNPs/ssDNA/miRNA-141 (d). Voltammograms were obtained in 5 mM (K_3_Fe(CN)_6_^4−/3−^) + 0.1 M KCl with a scan rate of 100 mV s^−1^.

The electrochemical performance of the GIPE-based system at various modification stages was evaluated via CV using 5 mM [K_3_Fe(CN)_6_]^3−/4−^ in 0.1 M KCl (Figure 2) as a redox probe. The bare GIPE exhibited an initial peak oxidation current of 157.2 μA (Curve a). Following the electrodeposition of AuNPs, the oxidation current increased significantly to 174.8 μA (Curve b), indicating enhanced electron transfer attributed to the enlarged electroactive surface area and the improved conductivity of the AuNP layer. Subsequent functionalization with 0.75 μM of the thiolated ssDNA-141 probe resulted in a decrease in the peak oxidation current to 125.8 μA (Curve c), confirming the successful immobilization of the probe layer and increased surface resistance due to ssDNA coverage. Upon hybridization with 100 pM target miRNA-141 for 15 min, the oxidation current further decreased to 87.0 μA (Curve d), indicating the successful hybridization and further obstruction of electron transfer. Using the Randles–Ševčík equation, we calculated the electroactive surface area to be 1.38 × 10^−4^ cm^2^ for the GIPE system, which increased to 1.53 × 10^−4^ cm^2^ for the GIPE/AuNPs system. This increase showcases the modifications of the GIPE system with AuNPs, which enhances its capacity for electron transfer during electrochemical reactions, thereby amplifying signal output.

**Figure 3:**
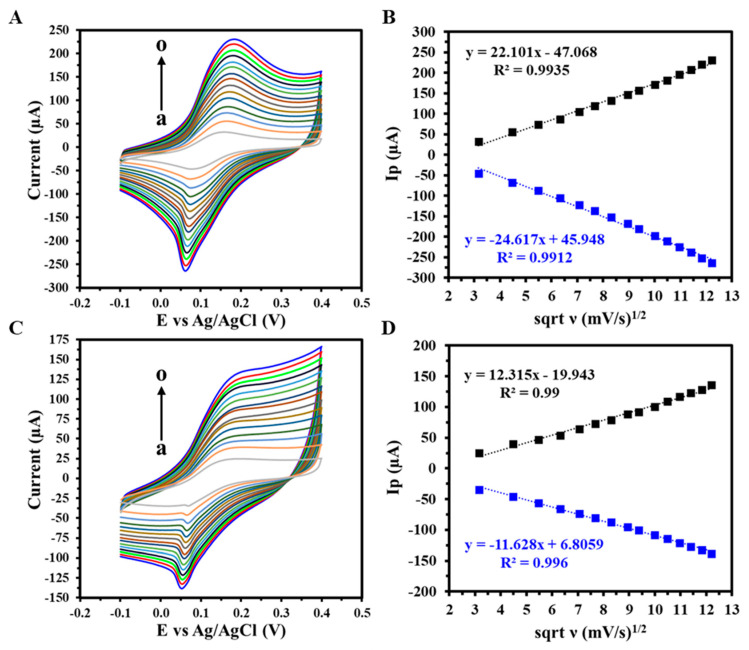
CV response of (**A**) GIPE/AuNPs and (**C**) GIPE/AuNPs/ssDNA from varying scan rates (a–o: 10–150 mV s^−1^), with (**B**,**D**) a corresponding linearity plot of peak anodic and cathodic current densities vs. the square root of scan rates (ν). Voltammograms were obtained in 5 mM (K_3_Fe(CN)_6_^4−/3−^) + 0.1 M KCl.

CV was employed to investigate the electron transfer mechanism of the GIPE/AuNPs and GIPE/AuNPs/ssDNA-modified electrodes over a range of scan rates (10–150 mV s^−1^) using 5 mM [K_3_Fe(CN)_6_]^3−/4−^ in 0.1 M KCl, as shown in Figure 3A and Figure 3C, respectively. The peak oxidation and reduction currents increased with increasing scan rates. The corresponding linear regression analyses between the peak currents and the square root of the scan rate (ν^1/2^) (Figure 3B,D) confirmed that the redox process at both electrode interfaces is a diffusion-controlled electron transfer process [39]. Figure 3B,D produced high correlation coefficients equal to or greater than R^2^ = 0.99 for anodic and R^2^ = 0.99 for cathodic currents, respectively, confirming the stability and reproducibility of electron transfer at the modified electrode surfaces.

### 3.2. Analytical Detection of miRNA-141 with GIPE/AuNPs/ssDNA Biosensor

**Figure 4:**
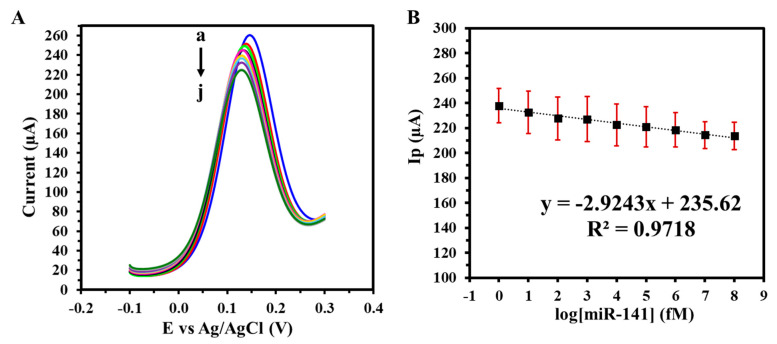
(**A**) SWVs of varying target miRNA-141 concentrations (a: 0 M, b: 1 fM, c: 10 fM, d: 100 fM, e: 1 pM, f: 10 pM, g: 100 pM, h: 1 nM i: 10 nM, j: 100 nM) in 1X PBS; (**B**) corresponding calibration curve. Voltammograms were obtained in 5 mM (K_3_Fe(CN)_6_^4−/3−^) + 0.1 M KCl. Experiments were conducted in duplicate.

The detection performance of the GIPE/AuNPs/ssDNA biosensor was assessed through hybridization experiments using miRNA-141 target solutions prepared in 1X PBS. Following incubation, the sample was aspirated, and the electrode underwent a rigorous washing protocol to eliminate unbound or nonspecifically bound nucleic acids. This consisted of two consecutive cycles of five sequential additions and removals of 100 μL of TE buffer by pipette. SWV was subsequently performed under optimized conditions to capture the biosensor’s electrochemical response corresponding to the hybridization event. The biosensor’s analytical performance was assessed using SWV in 1X PBS with target miRNA-141 concentrations ranging from 1 fM to 100 nM. As shown in Figure 4A, increasing target concentrations led to progressive decreases in the peak current, attributed to miRNA-141 hybridization and associated impedance at the electrode surface. The resulting calibration curve (Figure 4B) exhibited a linear relationship with a sensitivity of 41.37 fM µA^−1^ cm^−2^ and a calculated LOD of 12.27 fM, confirming the biosensor’s high sensitivity and dynamic response over a wide range of biologically relevant concentrations.

### 3.3. Selectivity, Stability, and Reproducibility Assessment

**Figure 5:**
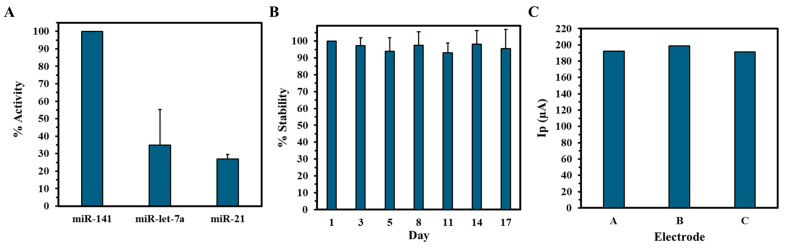
Selectivity and stability of the electrochemical biosensor. (**A**) Percent activity of miRNA-141 biosensor against non-complementary miRNAs. (**B**) Percent stability of GIPE/AuNPs/ssDNA vs. days of storage. Selectivity and stability experiments were conducted in duplicate. (**C**) Reproducibility of peak current (Ip) output of three separate GIPE/AuNPs/ssDNA systems.

Selectivity was evaluated by exposing the biosensor to non-target miRNAs, miRNA-21, and miRNA-let-7a at the same concentration (100 pM). As shown in Figure 5A, the relative current responses were markedly lower compared to miRNA-141, with current reductions in only 35% and 27%, respectively. These findings demonstrate the biosensor’s high selectivity for miRNA-141, likely due to strong complementary base pairing and minimized cross-reactivity. In contrast, the partial sequence mismatches with the non-target miRNAs hinder hybridization efficiency, resulting in weaker or transient interactions and subsequently reduced signal output change. The biosensor’s stability was also monitored over a 17-day storage period at 4 °C using SWV (Figure 5B). The biosensor retained approximately 96% of its initial electrochemical response, demonstrating robust long-term stability under appropriate storage conditions. This is attributed to the integration of biocompatible gold ink with electrodeposited AuNPs to enhance electrical conductivity and surface functionalization that ensures the structural stability of the electrodes under operational conditions. Reproducibility was achieved by analyzing the peak current output of three independent GIPE/AuNPs/ssDNA systems using SWV. The average current output of the GIPE/AuNPs/ssDNA systems was calculated to be 194.1 ± 3.2 µA, showcasing the excellent consistency of current output between different electrode systems.

### 3.4. Performance in Synthetic Urine Matrices

**Figure 6:**
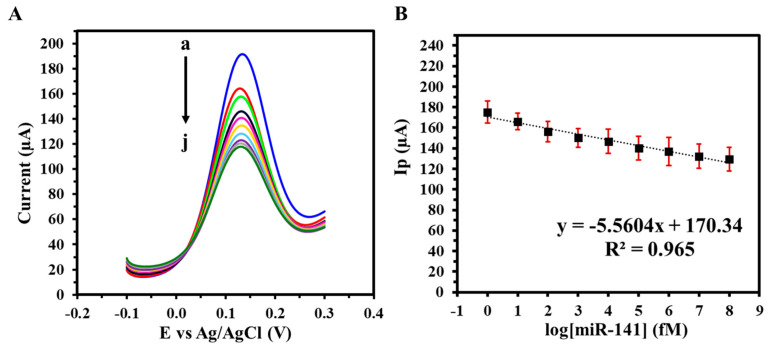
(**A**) SWVs of varying target miRNA-141 concentrations (a: 0 M, b: 1 fM, c: 10 fM, d: 100 fM, e: 1 pM, f: 10 pM, g: 100 pM, h: 1 nM i: 10 nM, j: 100 nM) in synthetic urine with (**B**) corresponding calibration curve. Voltammograms were obtained in 5 mM (K_3_Fe(CN)_6_^4−/3−^) + 0.1 M KCl. Experiments were conducted in duplicate.

To assess the GIPE/AuNPs/ssDNA biosensor’s translational potential, its performance was further validated in synthetic urine spiked with miRNA-141 at concentrations ranging from 1 fM to 100 nM. As illustrated in Figure 6A, the sensor maintained a concentration-dependent response comparable to that observed in PBS, with consistent peak current reductions across the tested range. The corresponding calibration curve in urine (Figure 6B) demonstrated excellent linearity, a lower LOD of 2.15 fM, and enhanced sensitivity of 78.66 fM µA^−1^ cm^−2^. The detection of miRNA-141 in synthetic urine matrices demonstrates the GIPE/AuNPs/ssDNA biosensor’s suitability for non-invasive testing, which is a critical attribute for patient-centric and decentralized healthcare models.

A comparative analysis (Table 2) highlights the superior performance of the GIPE/AuNPs/ssDNA biosensor in terms of detection range and sensitivity over previously reported electrochemical biosensors targeting urinary miRNAs. These studies span a diverse range of sensor platforms, including screen-printed carbon electrodes (SPCEs), single-walled carbon nanotube (SWNT) hybrids, graphene-based nanostructures, and indium tin oxide (ITO) interfaces, each functionalized with distinct probe strategies for targeting specific miRNA sequences. As shown in Table 2, several biosensors achieve good sensitivity, with LODs in the low femtomolar-to-attomolar range. Notably, the SPCE/MNB-CP/SiRP system for miR-124 detection reported an LOD of 0.65 fM, while a plasmonic-enhanced PS/AuNSs/ssDNA/2SMB platform targeting miRNA-200b achieved an LOD of 0.122 fM, currently among the lowest reported [39,40]. However, these approaches often involve complex fabrication processes, multistep labeling reactions, or extensive sample pre-treatment protocols, which may constrain their adaptability for rapid and low-cost POC deployment.

In contrast, the GIPE/AuNPs/ssDNA biosensor achieved a highly competitive LOD of 2.15 fM and an extensive linear detection range spanning from 1 fM to 100 nM, outperforming or matching several high-performance systems while maintaining a streamlined, label-free detection mechanism. For example, in comparison to the SPGE/AuNPs/DNA-MB sensor (LOD = 2 nM) [36] and the SPCE/SA/ssDNA platform (LOD = 17 fM) [41], the GIPE/AuNPs/ssDNA biosensor exhibited over 100- to 1000-fold greater sensitivity. Additionally, while the SWNTs/ssDNA system demonstrated a comparable detection range (10 fM–1 nM) [37], the GIPE/AuNPs/ssDNA biosensor extended the upper linear range by two orders of magnitude, enhancing its utility for clinical samples with variable biomarker expression levels. Overall, the combination of ultralow detection limits, broad linear dynamic range, simplified fabrication, and operational ease highlights the GIPE/AuNPs/ssDNA biosensor as a promising platform for miRNA-based diagnostics. Its performance is not only competitive with more complex and costly counterparts but also optimized for real-world implementation in decentralized healthcare environments.

## 4. Conclusions

We successfully developed and evaluated a GIPE/AuNPs/ssDNA electrochemical biosensor for the sensitive and selective detection of miRNA-141, a biomarker associated with aggressive prostate cancer. The use of inkjet printing technology enabled the precise deposition of AuNP-based conductive patterns onto flexible paper substrates, offering a low-cost, scalable, and rapid fabrication approach suitable for POC applications. Electrochemical measurements performed using SWV demonstrated excellent analytical performance over a broad dynamic range (1 fM to 100 nM), with a calculated sensitivity of 78.66 fM µA^−1^ cm^−2^ and an impressively low LOD of 2.15 fM. Importantly, the detection process was completed within 15 min, demonstrating the system’s potential for rapid diagnostics. Selectivity studies further confirmed the platform’s ability to discriminate miRNA-141 from non-complementary sequences, validating its clinical relevance for prostate cancer screening and monitoring. In terms of sample compatibility, the GIPE/AuNPs/ssDNA biosensor demonstrated reliable performance in synthetic urine matrices, validating its relevance for non-invasive detection workflows, thereby establishing a robust foundation for the design of next-generation, paper-based electrochemical biosensors that are affordable, disposable, and suitable for rapid, POC diagnostics. While this study establishes proof of concept for inkjet-printed gold electrodes in the electrochemical detection of synthetic miRNA targets, future work will focus on validating the platform using clinically relevant samples. This includes the detection of endogenous miRNA biomarkers in patient-derived fluids, such as blood serum or plasma, to assess the analytical performance in complex biological matrices. Such studies will be critical for evaluating the sensor’s diagnostic utility, specificity, and robustness under real-world conditions.

The continued refinement of the platform, through microfluidic integration, wireless data transmission, and clinical sample validation, may significantly enhance its translational potential. Ultimately, this approach offers a promising pathway toward accessible and equitable diagnostic technologies capable of improving early disease detection, treatment decision-making, and global health outcomes.

## Figures and Tables

**Table 1:** Oligonucleotide sequences.

Name	Nucleotide Sequence
ssDNA-141 probe	5′-TCC AAC ACT GTA CTG GAA GAT-G/3ThioMC3-D/3′
miRNA-141	5′-CAU CUU CCA GUA CAG UGU
miRNA-21	5′-UAG CUU AUC AGA CUG AUG
miRNA-let7a	5′-UGA GGU AGU AGG UUG UAU

**Table 2:** Recent electrochemical biosensors for detection of urinary miRNAs.

No.	Sensor Platform	Sample Type	Linear Range	LOD	Ref.
1	SPCE/MNB-CP/SiRP	miR-124 extracted from urine and resuspended in 1X PBS	1 fM–100 nM	0.65 fM	[40]
2	SPGE/AuNPs/DNA-MB	miRNA-21 spiked in urine + NaCl	50–340 nM	2 nM	[41]
3	SWNTs/ssDNA	miRNA-21 extracted from urine and 40-fold diluted in PBS	10 fM–1 nM	3 fM	[42]
4	SPCE/sABP/FITC-miRNA-141/HRP-TMB	miRNA-141 extracted from urine and diluted to 218 nM in elution buffer	1 pM–10 nM	0.1 pM	[43]
5	GNO/PNA	miRNA-21, miRNA-1246, and let-7b in urine	10 fM–10 nM	10 fM	[44]
6	SPCE/SA/ssDNA	miRNA-21 spiked in protein and other macromolecule-free urine	10 fM–10 nM	17 fM	[45]
7	ITO/TDNA	miRNA-21 in urine	10 fM–1 nM	10 fM	[46]
8	PS/AuNSs/ssDNA/2SMB	miRNA-200b from lysed exosomes in concentrated urine samples	100 aM–100 pM	0.122 fM	[47]
9	GIPE/AuNPs/ssDNA	miRNA-141 spiked in synthetic urine	1 fM–100 nM	2.15 fM	This Work

SPGE: screen-printed gold electrode; MB: methylene blue; SWNTs: single-walled carbon nanotubes; SPCE: screen-printed carbon electrode; sABPs: streptavidin–biotin probes; FTIC: fluorescein-modified detection probe; HRP: horseradish peroxidase; TMB: tetramethylbenzidine; MNB-CPs: capture probe labeled magnetic bead particles; SiRPs: silica particles loaded with methylene blue reporter probes; GNO: graphene oxide nanosheet; PNAs: peptide nucleic acids; SA: naphthalene sulfonic acid; ITO: iridium tin oxide; TDNA: catapult tetrahedral DNA; PSs: polystyrene sheets; AuNSs: gold nanostars; 2SMB: two-step hybridization using methylene blue-tagged signaling barcode.

## Data Availability

Data is contained within the article or Supplementary Materials.

## Supplementary Materials

The following supporting information can be downloaded at: https://www.mdpi.com/article/10.3390/bios15060364/s1, Figure S1: GIPE array and system design. (A) Photograph of the gold inkjet-printed 48-electrode system array on a 17.5 cm × 12.5 cm photopaper. (B) Schematic of completed GIPE system fabrication. WE: working electrode; CE: counter electrode: RE: reference electrode.
