# Supplementary material for: Electrochemical Detection of Prostate Cancer—Associated miRNA-141 Using a Low-Cost Disposable Biosensor
Source: Biosensors (Basel). 2025 Jun 6;15(6):364. doi: 10.3390/bios15060364 (PMC12190380; doi:10.3390/bios15060364)
Supplement: Supplementary file 1 [file biosensors-15-00364-s001.zip › biosensors-3653300-supplementary.pdf]

Supplementary data

Electrochemical Detection of Prostate Cancer–Associated miRNA-141 Using a Low-Cost Disposable Biosensor

Alexander Hunt[1] and Gymama Slaughter[1,2*]

[1]*Center for Bioelectronics, Old Dominion University, Norfolk, VA- 23508, USA*
[2]*Department of Electrical and Computer Engineering, Old Dominion University, Norfolk, VA- 23508, USA*

*gslaught@odu.edu

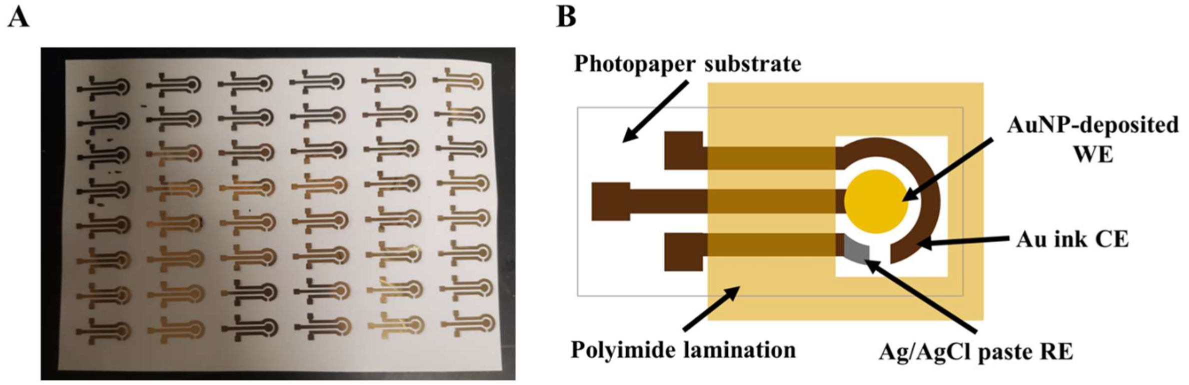

Figure S1. GIPE array and system design. (A) Photograph of the gold inkjet-printed 48-electrode system array on a 17.5 cm × 12.5 cm photopaper. (B) Schematic of completed GIPE system fabrication. WE: working electrode; CE: counter electrode: RE: reference electrode.
